# A metabolic map of hematopoietic stem cells

**DOI:** 10.1186/2049-3002-2-S1-P1

**Published:** 2014-05-28

**Authors:** Michalis Agathocleous, Zeping Hu, Sean Morrison

**Affiliations:** 1 Children’s Research Institute, UT Southwestern Medical Center, Dallas, TX, USA

## Background

A general problem in biology is whether different types of cells in the same tissue are metabolically different from each other, and whether such differences are important for cellular function. Experiments that can comprehensively measure the cellular metabolome typically require millions of cultured cells and cannot be used with small numbers of rare cells freshly isolated from tissues. In particular, hematopoietic stem cells (HSCs), the blood forming stem cells of the bone marrow, have been intensively studied for decades but their metabolic composition is largely unknown.

## Results

We have developed a method to measure metabolites in small numbers of HSCs. The HSC isolation and mass spectrometry methods have been optimized for maintenance of the metabolome during purification, sensitivity and robustness. About 50 metabolites can be quantified from 10,000 HSCs, covering a wide spectrum of the cellular metabolome. Several metabolic differences exist between HSCs and other bone marrow cells, including restricted progenitors. I am investigating the role of these metabolic adaptations in HSCs.

## Conclusion

The ability to profile the metabolome of rare cells isolated directly from tissues opens the possibility to metabolically compare stem cells to other purified populations of cells at different stages of differentiation, to test the metabolic consequences of physiological challenges like aging on specific populations of cells, and to test whether other rare cells, like subpopulations of cancer cells from the same tumor, are metabolically distinctive.

